# Commentary: The effects of perioperative dexmedetomidine infusion on hemodynamic stability during laparoscopic adrenalectomy for pheochromocytoma: a randomized study

**DOI:** 10.3389/fmed.2024.1353046

**Published:** 2024-04-08

**Authors:** Beibei Zhang, Xiaodong Wang, Yingchao Guan, Minghao Chen

**Affiliations:** Department of Anesthesiology, Weihai Municipal Hospital, Cheeloo College of Medicine, Shandong University, Weihai, China

**Keywords:** pheochromocytoma, hemodynamics, vasoactive drugs, confounding factors, baseline blood pressure

## 1 Introduction

As is widely acknowledged, resection of pheochromocytoma often results in significant hemodynamic changes in patients. However, maintaining hemodynamic stability is a paramount goal for every anesthesiologist. Therefore, the prudent and effective use of anesthesia and vasoactive drugs presents a significant challenge. We were particularly intrigued by the study entitled “*The effects of perioperative dexmedetomidine infusion on hemodynamic stability during laparoscopic adrenalectomy for pheochromocytoma: a randomized study*” (1) and would like to share our insights.

The authors evaluated the impact of intraoperative administration of dexmedetomidine (0.5 μg/kg/h) on the blood pressure and heart rate of patients with pheochromocytoma undergoing laparoscopic adrenalectomy. The study found that dexmedetomidine was associated with a significant reduction in the maximum blood pressure (control group vs. dexmedetomidine group: 182 ± 31 vs. 161 ± 20, 102 ± 17 vs. 90 ± 10, and 128 ± 22 vs. 116 ± 12 [mean ± SD] mmHg and *P* = 0.020, 0.015, and 0.040 for systolic, diastolic, and mean blood pressure, respectively) and heart rate (control group (108 ± 15 bpm) vs. dexmedetomidine group (95 ± 12 bpm), *P* = 0.010). These findings showed the role of dexmedetomidine in preserving intraoperative hemodynamic stability. Subsequently, the authors indicated that dexmedetomidine is a promising adjunct medication to general anesthesia for patients undergoing laparoscopic adrenalectomy for pheochromocytoma. The authors noted that certain vasoactive drugs, such as nitroprusside, esmolol, and norepinephrine, could markedly influence the primary outcome measures of this study, including blood pressure and heart rate. In Table 2 of the mentioned article, we observed substantial variability in the dosage of esmolol administered to individual patients, yet the authors did not furnish detailed information regarding the standard, timing, method of administration, and cessation of these drugs. Moreover, within this section, the authors solely performed a statistical analysis on the frequency of these drugs' usage in the experimental and control groups, which may not sufficiently elucidate their impact on the study's primary outcome measures. Consequently, the potential influence of these vasoactive drugs on the study's primary outcome indicators cannot be ignored. Furthermore, additional statistical analyses on the relevant data are imperative, including propensity score matching (2) and multivariate adjustment analysis, to mitigate the impact of these confounding factors on the study results and enhance the reliability of the experimental findings.

In this study, although all patients received oral phenoxybenzamine (dose: 60 mg per day, range: 20–100 mg per day) for 3 weeks before surgery according to the hospital protocol, hemodynamic instability occurred during tumor manipulation, requiring administration of low-dose esmolol, sodium nitroprusside, or nicardipine. The study indicated that the administration of dexmedetomidine in catecholamine surge situations appeared to effectively attenuate maximal blood pressure and successfully manage sudden hypertensive crises. Furthermore, in this study, the authors defined the blood pressure measured before anesthesia induction in the supine position in the operating room as the baseline blood pressure, which is a non-invasive measurement. However, during the operation, the recorded blood pressure was obtained invasively. Extensive evidence, along with clinical practice, consistently indicated that invasive blood pressure readings tend to be higher than non-invasive measurements (3). In addition, the authors did not specify the site of arterial puncture for invasive blood pressure measurement, despite the diverse options in clinical practice, such as the radial artery, dorsal foot artery, and femoral artery, each yielding different values (4). This lack of detail introduces the possibility of bias in the study results, potentially leading to conclusions that diverge from the actual findings.

As the authors did not assess the side effects, it is noteworthy that dexmedetomidine, a medication used for sedation, can lead to various side effects that may occur in any patient population, including those with pheochromocytoma. These side effects include hypotension, which can be of concern in patients with pheochromocytoma due to the tumor's effects on blood pressure, as well as bradycardia. Additionally, sedation from dexmedetomidine can lead to respiratory depression or airway obstruction, while dry mouth and gastrointestinal upset, such as nausea and vomiting, are also possible.

The authors' exploration of dexmedetomidine's role in anesthesia maintenance during pheochromocytoma resection holds substantial clinical importance. To enhance the study's credibility and reproducibility, it is crucial for the authors to provide more comprehensive details regarding the administration of vasoactive drugs. Addressing and elucidating the differences between invasive and non-invasive blood pressure measurements, along with an improved data analysis methodology, would significantly contribute to strengthening the study's integrity and its applicability in clinical settings.

## Author contributions

BZ: Writing – original draft. XW: Writing – review & editing. YG: Writing – review & editing. MC: Writing – review & editing.
